# Enigma proteins regulate YAP mechanotransduction

**DOI:** 10.1242/jcs.221788

**Published:** 2018-11-22

**Authors:** Ahmed Elbediwy, Hannah Vanyai, Maria-del-Carmen Diaz-de-la-Loza, David Frith, Ambrosius P. Snijders, Barry J. Thompson

**Affiliations:** 1 Epithelial Biology Laboratory, The Francis Crick Institute, 1 Midland Rd, London NW1 1AT, UK; 2 Mass Spectrometry Science Technology Platform, The Francis Crick Institute, 1 Midland Rd, London NW1 1AT, UK

**Keywords:** Enigma, PDLIM5, PDLIM7, Hippo, Integrin, YAP, Mechanotransduction

## Abstract

Human cells can sense mechanical stress acting upon integrin adhesions and respond by sending the YAP (also known as YAP1) and TAZ (also known as WWTR1) transcriptional co-activators to the nucleus to drive TEAD-dependent transcription of target genes. How integrin signaling activates YAP remains unclear. Here, we show that integrin-mediated mechanotransduction requires the Enigma and Enigma-like proteins (PDLIM7 and PDLIM5, respectively; denoted for the family of PDZ and LIM domain-containing proteins). YAP binds to PDLIM5 and PDLIM7 (hereafter PDLIM5/7) via its C-terminal PDZ-binding motif (PBM), which is essential for full nuclear localization and activity of YAP. Accordingly, silencing of PDLIM5/7 expression reduces YAP nuclear localization, tyrosine phosphorylation and transcriptional activity. The PDLIM5/7 proteins are recruited from the cytoplasm to integrin adhesions and F-actin stress fibers in response to force by binding directly to the key stress fiber component α-actinin. Thus, forces acting on integrins recruit Enigma family proteins to trigger YAP activation during mechanotransduction.

This article has an associated First Person interview with the first author of the paper.

## Abstract (precis)

**Summary:** The Enigma family proteins (PDLIM5/7) link YAP with F-actin stress fibers to participate in integrin-mediated mechanotransduction.

## INTRODUCTION

Integrin adhesion to the extracellular matrix is a fundamental mechanism controlling tissue growth and form during normal development (Wickstrom et al., 2011) and in cancer (Hamidi et al., 2016). In addition to providing adhesion to the matrix, integrins enable cells to sense mechanical forces to activate ‘inside-out signaling’, which stimulates integrin binding to matrix ligands, as well as ‘outside-in signaling’, which activates cytoplasmic mechanotransduction pathways to regulate cell behavior (Legate et al., 2009; Ross et al., 2013; Sun et al., 2016). In particular, focal adhesion kinase (FAK; also known as PTK2) and Src family kinases have important roles in integrin signaling (Avizienyte and Frame, 2005) and in synergy between integrin and growth factor signaling (Chen et al., 2018). Recent work has unveiled a consensus integrin adhesome containing a large number of proteins that are likely to be involved in either adhesion or mechanotransduction (Horton et al., 2015).

One crucial downstream effector of integrin signaling is the Yes-associated protein (YAP, also known as YAP1), originally discovered by virtue of its ability to form a complex with the Src family kinase, Yes (Sudol, 1994). YAP (and its paralog TAZ, also known as WWTR1) was found to be a transcriptional co-activator that is negatively regulated by interaction with 14-3-3 proteins (after serine phosphorylation) and positively regulated by interaction with PDZ domains (via a C-terminal PDZ-binding motif or PBM) (Kanai et al., 2000; Yagi et al., 1999). YAP was subsequently shown to function as a co-activator for the TEAD family of DNA-binding transcription factors, even though the majority of the YAP protein was localized to the cytoplasm in complex with 14-3-3 proteins (Vassilev et al., 2001). Although YAP is cytoplasmic at high cell density, it can translocate to the nucleus when cells lose contact with one another and/or spread out across their substrate (Zhao et al., 2007). Importantly, the nuclear localization of YAP was shown to require the presence of the C-terminal PDZ-binding motif (Oka and Sudol, 2009). YAP shuttles dynamically between the cytoplasm and nucleus, with its bulk distribution likely to be determined by relative binding to cytoplasmic (e.g. 14-3-3) versus nuclear (e.g. TEAD) proteins (Badouel et al., 2009; Ren et al., 2010; Vassilev et al., 2001; Zhang et al., 2008; Zhao et al., 2007), or possibly through regulated nuclear import or export (Ege et al., 2018; Furukawa et al., 2017; Manning et al., 2018).

Culture of cells on micropatterns and different matrix types suggested that the size of the contact cells make upon spreading over their basal substrate, substrate stiffness and the resulting mechanical tension on F-actin stress fibers are key determinants of YAP subcellular localization in response to cell density (Dupont et al., 2011; Wada et al., 2011). Nevertheless, different groups have drawn opposite conclusions as to whether mechanical tension on stress fibers signals via the Hippo pathway kinases LATS1 and LATS2 (hereafter LATS1/2) to control YAP localization, or via a LATS1/2-independent pathway (Dupont et al., 2011; Meng et al., 2018; Wada et al., 2011). Recent work has confirmed a key role for integrin adhesion to the extracellular matrix and integrin signaling via talin, Rho and Src family kinases as important mechanosensory mechanisms that regulate YAP (Elbediwy et al., 2016; Elosegui-Artola et al., 2016; Kim and Gumbiner, 2015; Tang et al., 2013). Integrin–Src signaling can affect LATS1/2-mediated phosphorylation of YAP (Elbediwy et al., 2016; Kim and Gumbiner, 2015) possibly via direct tyrosine phosphorylation of LATS1 (Si et al., 2017), or via crosstalk with growth factor signaling (Fan et al., 2013; Kim and Gumbiner, 2015). Alternatively, integrin–Src signaling can also activate YAP via direct tyrosine phosphorylation of YAP in its transcriptional activation domain (Li et al., 2016; Taniguchi et al., 2015). Finally, YAP can also sense mechanical stretching of E-cadherin-based adherens junctions (Benham-Pyle et al., 2015), possibly via Ajuba, LIMD1 and TRIP6-mediated LATS1/2 inhibition (Dutta et al., 2018; Ibar et al., 2018; Rauskolb et al., 2014), via Hippo kinase (MST1 and MST2) inactivation (Fletcher et al., 2018), or via Src activation at adherens junctions (Gomez et al., 2015; Kim et al., 2011; McLachlan et al., 2007; Roura et al., 1999; Serrels et al., 2011; Shindo et al., 2008; Tsukita et al., 1991). How YAP might be recruited to integrin (or E-cadherin) adhesions in order to be directly phosphorylated by Src family kinases in response to mechanical force is an important unsolved problem.

## RESULTS AND DISCUSSION

**Fig. 1.**
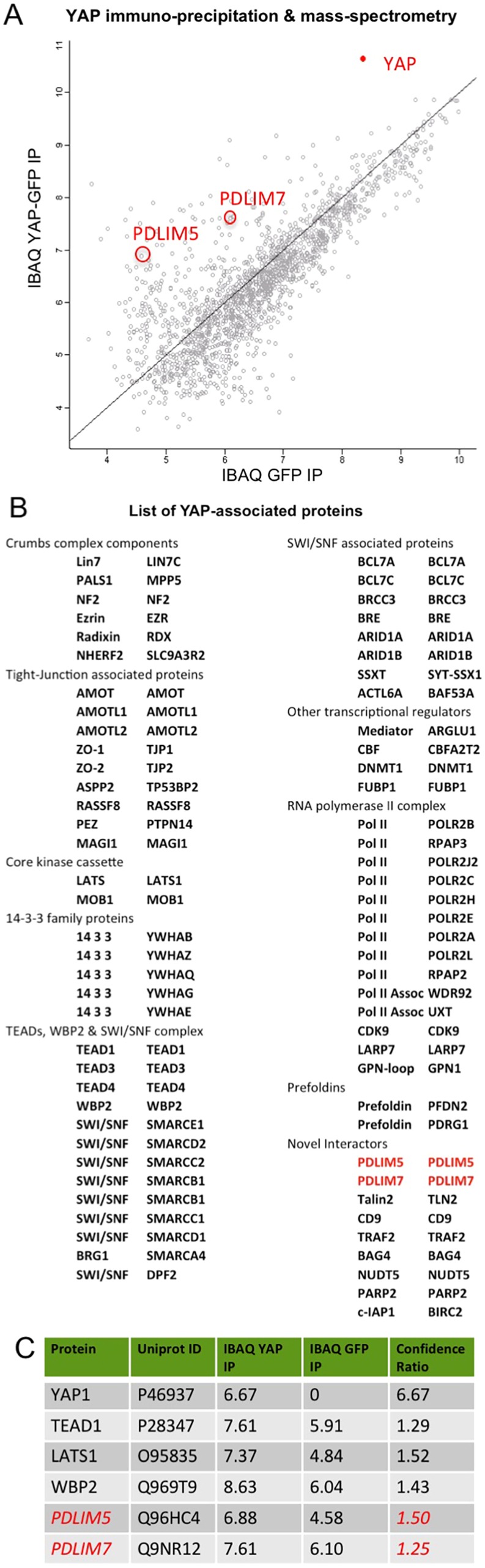
**YAP immunoprecipitation and mass-spectrometry analysis of binding partners.** (A) YAP IP-MS analysis of co-precipitated proteins identifies the Engima family proteins PDLIM5 and PDLIM7 as novel YAP-associated proteins. Axes are log_10_-transformed values. (B) List of all YAP-associated proteins identified in the experiment shown in A. (C) Comparison of confidence ratios for PDLIM5/7 and known YAP interactors.

**Fig. 2.**
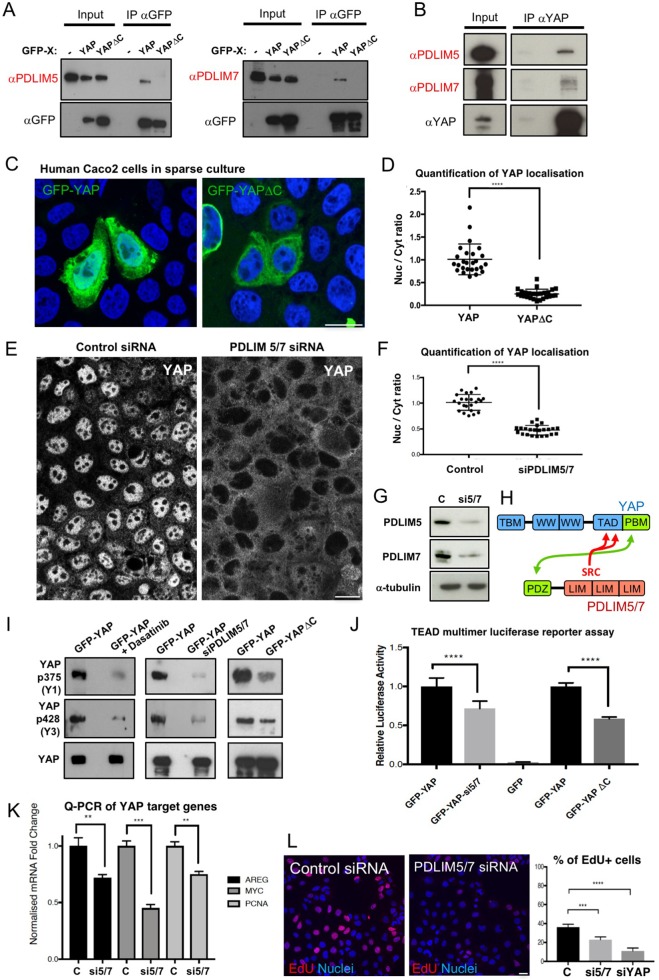
**The Enigma family proteins PDLIM5/7 bind to the YAP C-terminal PBM and promote YAP nuclear localization and transcriptional activity.** (A) Confirmation of YAP–PDLIM5 and YAP–PDLIM7 interaction by co-immunoprecipitation (IP) of GFP-tagged YAP, and immunoblotting with anti-PDLIM5 and anti-PDLIM7 antibodies. Both Enigma family proteins PDLIM5 and PDLIM7 bind to the YAP C-terminal PBM, as deletion of this motif (YAPΔC) abolishes the interaction in co-immunoprecipitation experiments. (B) Endogenous YAP co-immunoprecipitates with PDLIM5 and PDLIM7. (C) Deletion of the C-terminal PBM (YAPΔC) reduces nuclear localization of GFP-tagged YAP in human Caco2 cells plated at low density. Results in A–C are representative of *n*=3 biological replicates. (D) Quantification (mean±s.d., *n*=3) of YAP localization for experiments as shown in C. ****P<0.001 (Student's *t*-test). (E) Double-silencing of both PDLIM5 and PDLIM7 expression in human Caco2 cells strongly reduces nuclear localization of YAP. Results are representative of *n*=6 biological replicates. (F) Quantification (mean±s.e.m.) of YAP localization for experiments as shown in E. ****P<0.001 (Student's *t*-test). (G) Confirmation of depletion of PDLIM5 and PDLIM7 expression levels for the siRNA (si5/7) treatment used in E. C, control siRNA. Results are representative of *n*=3 biological replicates. (H) Schematic diagram of the interaction between the YAP PBM and the PDZ domain of PDLIM5/7 proteins. Proximity of Src phosphorylation sites to the PBM is shown. (I) Silencing of PDLIM5/7 in human Caco2 cells reduces tyrosine phosphorylation of YAP. Results are representative of *n*=3 biological replicates. (J) Silencing of PDLIM5/7 in human Caco2 cells reduces YAP-driven TEAD transcriptional activity, as measured by a TEAD multimer luciferase reporter assay (relative to a *Renilla* luciferase control) (mean±s.e.m., *n*=10 from two independent experiments). Similar results were obtained upon deletion of the YAP C-terminal PBM. ****P<0.001 (Student's *t*-test). (K) Silencing of PDLIM5/7 in human Caco2 cells reduces expression of the YAP-target genes *AREG*, *MYC* and *PCNA* (mean±s.e.m., *n*=3). **P<0.01, ***P<0.005 (Student's *t*-test). (L) Silencing of PDLIM5/7 in human Caco2 cells reduces the rate of cell proliferation, as measured through a pulse of EdU incorporation. Upon loss of PDLIM5/7, proliferation slows by approximately half compared with control cells, which is comparable in magnitude to that achieved by silencing of YAP itself (siYAP). Results in the graph are mean±s.d. (*n*=3). ****P<0.001, ***P<0.005 (Student's *t*-test). Scale bars: 20 µm (C,E,L).

To identify possible binding partners of YAP, we performed immunoprecipitation (IP) and mass spectrometry (IP-MS) of GFP-tagged human YAP transfected into human HEK293T cells. Aside from many known interactors, which confirm the quality of our IP-MS analysis, we identified novel interactors including the PDZ and LIM domain-containing (PDLIM) family proteins Enigma (PDLIM7) and Enigma-like (PDLIM5) (Fig. 1A–C). Note that both Enigma family proteins were identified as members of the integrin adhesome, although their function remains poorly understood (Horton et al., 2015). We confirmed this interaction by co-IP of GFP–YAP and western blotting for the Enigma family proteins PDLIM5 and PDLIM7 (hereafter PDLIM5/7) (Fig. 2A). Importantly, deletion of the YAP PBM motif (YAPΔC) abolished the interaction between YAP and PDLIM5/7, suggesting that PDLIM5/7 bind to YAP via the PBM motif (Fig. 2A). We further find that IP of endogenous YAP also pulls down endogenous PDLIM5/7 (Fig. 2B). We confirmed previous observations (Oka and Sudol, 2009; Shimomura et al., 2014) that the PBM motif is important to promote YAP nuclear localization by comparison of the localization of transfected GFP–YAP with GFP–YAPΔC in sparsely plated human Caco2 epithelial cells (Fig. 2C,D).

To test whether Enigma family proteins are required for YAP to localize to the nucleus upon cellular stretching, we plated human Caco2 cells at a moderate density, and transfected them with either control or PDLIM5/7-targeted siRNAs. We find that control siRNAs had no effect, while silencing of the Enigma family proteins strongly inhibited nuclear localization of YAP, without affecting cell shape or density (Fig. 2E–G). Silencing of PDLIM5/7 also reduced the Src family kinase-dependent phosphorylation of YAP on Y375 and Y428, similar to what is seen upon loss of the YAP C-terminal PBM, without affecting the levels of phosphorylated Src (p-Src) or its localization to focal adhesions (Fig. 2H,I; Fig. S1). The reduction in YAP transcriptional activity upon siRNA knockdown of PDLIM5/7 was also comparable to that seen upon deleting the YAP C-terminal PBM in a TEAD-multimer reporter gene assay (Fig. 2J). Analysis of the YAP target genes *AREG*, *MYC* and *PCNA* by quantitative PCR (qPCR) revealed a comparable reduction in target gene expression upon silencing of PDLIM5/7 (Fig. 2K). Furthermore, the rate of cell proliferation, as measured from a pulse of EdU incorporation in cells undergoing S-phase of the cell cycle, is reduced upon silencing of PDLIM5/7 (Fig. 2L). We conclude that Enigma family proteins bind directly to YAP via the C-terminal PBM to promote YAP tyrosine phosphorylation, nuclear localization and transcriptional activation in human cells.

**Fig. 3.**
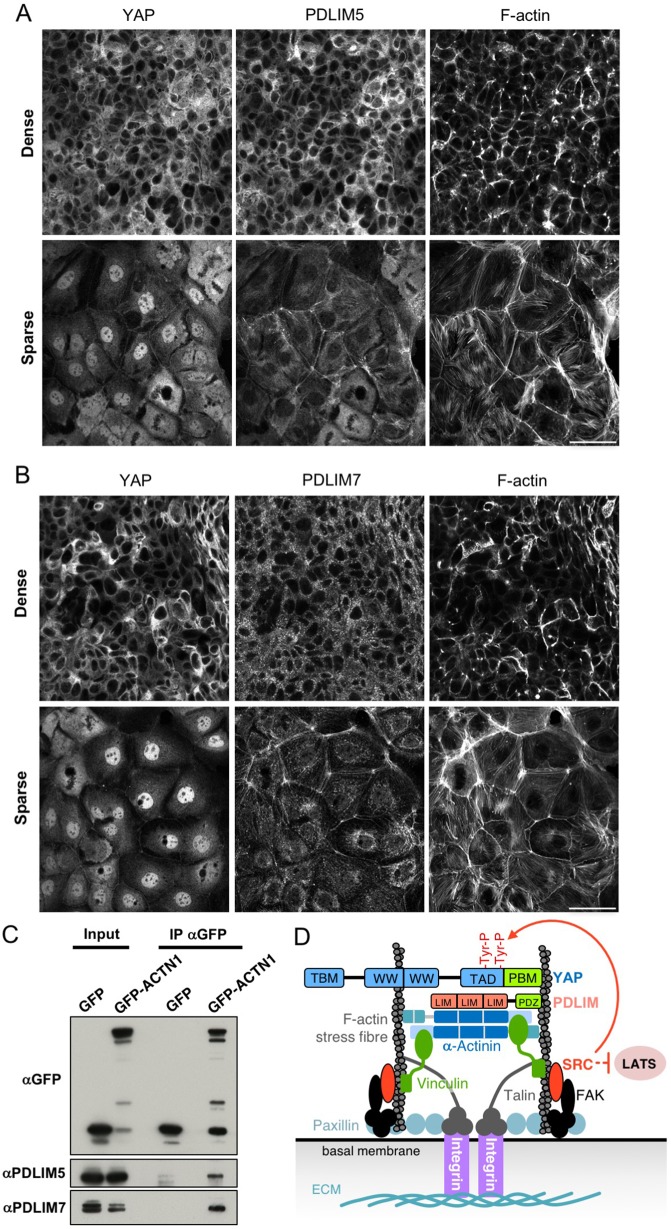
**PDLIM5/7 proteins localize in the cytoplasm in dense cells but to basal stress fibers in sparse cells and bind directly to the stress fiber component α-actinin 1.** (A) PDLIM5 localizes to the cytoplasm in dense cells but translocates, in part, to F-actin stress fibers and adherens junctions in sparsely plated cells. (B) PDLIM7 localizes to the cytoplasm in dense cells but translocates in part to F-actin stress fibers and adherens junctions in sparsely plated cells. (C) Co-immunoprecipitation of PDLIM5/7 proteins with GFP-tagged α-actinin 1 from human Caco2 cells. Results are representative of *n*=3 biological replicates. (D) Schematic diagram of YAP recruitment via the Enigma PDLIM5/7 proteins to integrin–Src signaling complexes to sense mechanical forces basally. Scale bar: 20 µm (A,B).

We next examined the subcellular localization of PDLIM7 and PDLIM5. We find that both proteins localize to the cytoplasm in densely cultured cells, similar to what is seen for YAP (Fig. 3A,B). In response to spreading of the cells upon plating at low density, both Enigma family proteins relocalize in part to F-actin stress fibers and focal adhesions, as well as to F-actin fibers at adherens junctions (Fig. 3A,B). At the same time, YAP translocates to the nucleus, suggesting that the relocalization of Enigma proteins to sites of mechanical force sensing could be a trigger for YAP nuclear localization (Fig. 3A,B). Accordingly, PDLIM5/7 can both be detected in a complex with the F-actin stress fiber component α-actinin 1 (ACTN1; Fig. 3C,D). We propose that, upon mechanical stimulation of human cells, Enigma family proteins bind to α-actinin 1 on F-actin stress fibers at integrin focal adhesions in order to promote tyrosine phosphorylation of YAP by Src family kinases, and thus YAP activation (Fig. 3D). Note that we find similar co-regulation of YAP and Enigma when cells are mechanically stimulated by plating on matrices of varying stiffness (Fig. S2). Interestingly, Src family kinases can also be activated at α-actinin-containing F-actin cables organized by E-cadherin-containing adherens junctions (McLachlan et al., 2007), and we are also able to detect some PDLIM5/7 localization with p-Src at adherens junctions as well as at focal adhesions (Fig. S3).

**Fig. 4.**
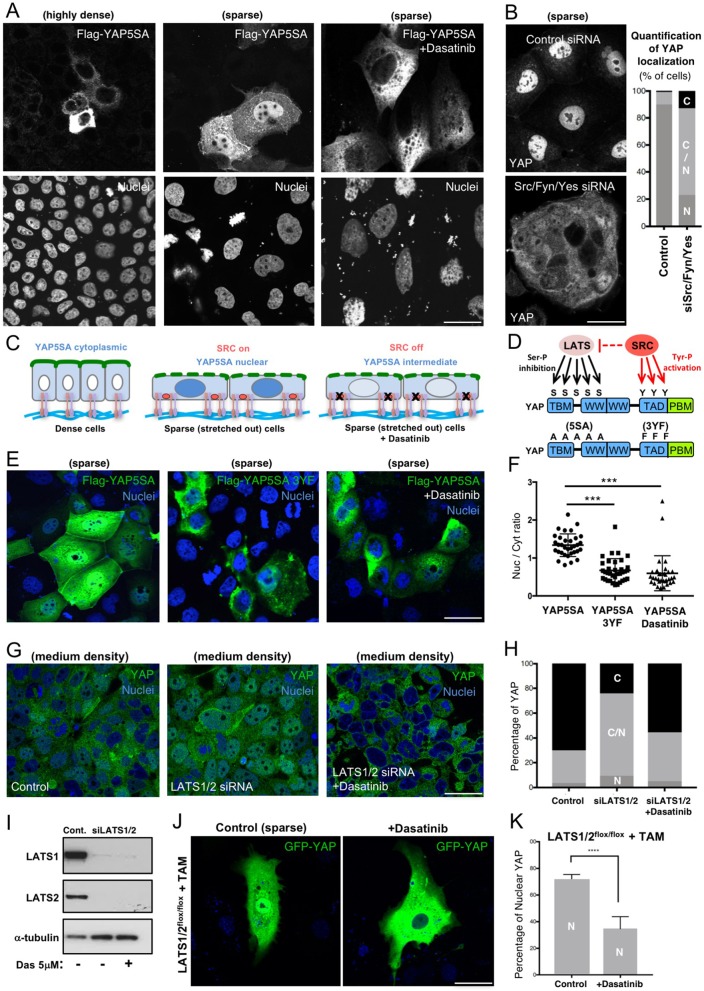
**Mechanical control of YAP can occur independently of the canonical LATS phosphorylation on serine residues seen in the Hippo pathway and involves Src family kinase phosphorylation on tyrosine residues.** (A) Mutation of five serine residues in YAP (5SA), to render it not capable of being phosphorylated by LATS kinase, does not lead to constitutively nuclear localization in human Caco2 cells. Flag-tagged YAP5SA responds to changes in cell density, becoming cytoplasmic in highly dense cultures and nuclear in sparse cultures with spread-out cells. The nuclear localization of YAP5SA is dependent on Src family kinases, as it is reduced upon treatment with the Src inhibitor Dasatinib. Results are representative of *n*=4 biological replicates. (B) Silencing of Src/Fyn/Yes kinases by triple siRNA causes a reduction in YAP nuclear localization. A quantification of the percentage of cells with each localization category (N, nuclear; N/C, nuclear and cytoplasmic; C, cytoplasmic) is shown on the right, *n*=3. (C) Schematic diagram of YAP5SA subcellular localization (blue) in response to cell density and upon treatment with Dasatinib in (A). Basal integrin attachments to the ECM are shown. (D) Schematic diagram of YAP phosphorylation by LATS1/2 and Src family kinases, and of serine (S) to alanine (A), and tyrosine (Y) to phenylalanine (F) mutations. (E) Flag-tagged YAP5SA nuclear localization depends upon three tyrosine residues in its transcriptional activation domain (TAD), whose mutation to phenylalanine (3YF) reduces nuclear localization in a manner similar to that seen upon treatment with Dasatinib. Results are representative of *n*=3 biological replicates. (F) Quantification of results from the experiment in E (mean±s.e.m., *n*=3). (G) YAP immunostaining (green) of human Caco2 epithelial cells at medium density transfected with either scrambled control siRNAs or LATS1/2 siRNAs (siLATS1/2) in the presence or absence of the Src family kinase inhibitor Dasatinib. DAPI marks nuclei (blue). Results are representative of *n*=3 biological replicates. (H) Quantification of results from the experiment in G (*n*=3). (I) Confirmation that LATS1/2 siRNAs, as used in experiment in G, effectively reduce LATS1 and LATS2 protein levels. (J) LATS1/2 double floxed MEFs transfected with Cre-ERt and YAP before treatment with Tamoxifen and Dasatinib to induce deletion of both LATS1 and LATS2 genes. (K) Quantification of YAP nuclear localization in experiments as in J (mean±s.e.m.; *n*=2 biological replicates each counting at least 600 cells from 8–10 independent areas over many coverslips). ****P<0.001 (Student's *t*-test). Scale bars: 20 μm (B), 30 μm (A,E), 50 μm (G,J).

We sought to test our hypothesis that Enigma proteins can induce nuclear localization of YAP by promoting direct phosphorylation of YAP by Src family kinases, rather than through an indirect mechanism that requires the phosphorylation of YAP by LATS family kinases. To test this idea, we examined the localization of phosphorylation mutant YAP5SA (with the five serine residues in YAP mutated in alanine residues), which is unable to be inhibited by canonical Hippo–LATS1/2 kinase signaling. We find that human YAP5SA remained cytoplasmic in densely cultured cells and became nuclear in sparsely cultured cells, indicating the requirement for a parallel pathway that regulates YAP nucleo-cytoplasmic translocation independently of canonical Hippo signaling (Fig. 4A). Treatment with the Src family kinase inhibitor Dasatinib, or PDLIM5/7 siRNA, reduced nuclear localization of YAP5SA, such that most cells had a comparable level of YAP5SA in both nucleus and cytoplasm – similar to the effect of Src/Fyn/Yes triple siRNA on endogenous YAP localization (Fig. 4A–C; Fig. S1). We next considered whether direct phosphorylation of YAP on multiple tyrosine residues by Src family kinases (Li et al., 2016) could account for density-dependent regulation of YAP5SA (Fig. 4D). Accordingly, we find that mutation of three tyrosine residues to phenylalanine (3YF) in the YAP transcriptional activation domain (TAD) reduces the nuclear localization of YAP5SA, similar to what was seen upon treatment with Dasatinib (Fig. 4D–F). Finally, Dasatinib also reduced YAP nuclear localization even in the absence of LATS1/2 induced by either siRNA silencing in densely cultured human epithelial cells (Fig. 4G–I) or by double-conditional knockout of homozygous floxed *LATS1^fl/fl^* and *LATS2^fl/fl^* upon ubiquitous expression of tamoxifen-inducible *Cre-ERt* allele together with GFP–YAP in mouse embryonic fibroblasts (Fig. 4J,K). These results suggest parallel regulation of YAP by LATS1/2 and Src family kinases in response to cell density, in agreement with recent findings in cholangiocarcinoma cells (Sugihara et al., 2018). Interestingly, we find overexpression of PDLIM proteins results in a moderate reduction in YAP localization (Fig. S4). This is consistent with the idea that PDLIM proteins act as a bridge between YAP and integrin adhesions, as overexpression would be predicted to saturate YAP in the cytoplasm and prevent productive association with integrin adhesions, thus reducing YAP tyrosine phosphorylation and nuclear localization.

In conclusion, our results indicate that mechanical stress-induced binding of Enigma proteins to α-actinin to basal stress fibers provides a platform for YAP to be tyrosine phosphorylated by Src family kinases to promote YAP nuclear localization and the full activation of YAP-driven transcription. Further work is necessary to understand how tyrosine phosphorylation promotes YAP localization to the nucleus, although such phosphorylation could promote either nuclear import, as in the case of STAT proteins (Reich, 2013; Reich and Liu, 2006), or interaction with nuclear proteins, such as TEADs or SWI/SNF components, which could maintain nuclear localization as well as regulate transcription (Skibinski et al., 2014; Song et al., 2017; Zhu et al., 2015). Importantly, this Enigma-dependent mechanism for regulation of YAP must act in parallel to inhibition of the canonical Hippo pathway initiated by integrin signaling, via multiple signaling pathways (Elbediwy et al., 2016; Elosegui-Artola et al., 2016; Kim and Gumbiner, 2015; Kissil et al., 2002; Meng et al., 2018; Sabra et al., 2017; Si et al., 2017; Xiao et al., 2002). It must also act in parallel to any direct mechanical regulation of the nucleus, which becomes strongly deformed and permeable to small proteins in extremely flattened cells (Elosegui-Artola et al., 2017; Shiu et al., 2018) and, indeed, Enigma proteins are no longer required for nuclear localization of YAP upon such extreme cellular flattening (data not shown). In future, it will be of great interest to investigate with genetically modified mice which of these mechanotransduction pathways operates in different mammalian tissues *in vivo*.

## MATERIALS AND METHODS

### Plasmids

pEGFP C3-YAP2 and pEGFP C3-YAP-DeltaC plasmids were Addgene plasmids #19055 and #21126 (deposited by Marius Sudol). pCMV-Flag YAP2 5SA was Addgene plasmid #27371 (deposited by Kun-Liang Guan). pEGFP C3-YAP2 3YF and pCMV-Flag YAP2 5SA 3YF were created using pEGFP C3-YAP2 and pCMV-Flag YAP2 5SA, respectively, by mutating the three tyrosine residues in question (Y375F Y391F and Y428F). Note the YAP constructs are generated using mRNA isoform 3. Site directed mutagenesis was performed by Creative Biogene. pEGFP-N1 α-actinin 1 was Addgene plasmid #11908 (deposited by Carol Otey). pNL2.2 - 8×TEAD and pRL-CMV Renilla were from Promega. All plasmids were transfected using Lipofectamine 3000 (Invitrogen). To constitutively overexpress human PDLIM7, the PDLIM7 (isoform 1) open-reading frame (ORF) was subcloned from the corresponding entry vector (Dharmacon; clone 3562 for PDLIM7 isoform 1) into the destination vector pcDNA-PDEST47 (Invitrogen, 12281010) by recombination using the Gateway LR clonase enzyme mix (Invitrogen,11791).

### Human cell culture

Human Caco-2 cells and HEK293T (Francis Crick Institute cell services) were grown in conditions as previously described (Elbediwy et al., 2016). All cells were subject to mycoplasma testing.

### Generation of LATS1/2 MEFs

All experiments were carried out in accordance with the United Kingdom Animal Scientific Procedures Act (1986) and UK Home Office regulations under project license number 70/7926. Mouse embryonic fibroblasts were derived from E14.5 *Lats1^lox/lox^*;*Lats2^lox/lox^* (Yi et al., 2016) carrying the *Rosa26-cre-ERT2* (Seibler et al., 2003) allele on a mixed background. At passage 4 or 5, 100,000 cells were plated in each well of an eight-well Ibidi chamber slide. At 24 h after plating, pEGFP C3-YAP2 was transfected using Lipofectamine 3000 while simultaneously adding tamoxifen. Transfection was left for a further 48 h and the medium was changed 24 h post tamoxifen treatment before cells were fixed and examined via a standard immunofluorescence protocol.

### Co-immunoprecipitation

HEK293T cells were transfected with the relevant plasmids using Lipofectamine 2000. The sample was then lysed and subjected to co-immunoprecipitation using a GFP Trap Kit containing lysis buffer (10 mM Tris-HCl pH 7.5, 150 mM NaCl, 0.5% NP-40 and 0.5 mM EDTA) (Chromotek). Lysis buffer was supplemented with PhosStop Phosphatase Inhibitor Cocktail Tablets (Roche), Protease Inhibitor Cocktail (Roche), 0.1 M NaF and 1 mM PMSF. Samples were left on ice to solubilize for 10 min, before being centrifuged (10000 r.p.m. for 10 min at 4°C), pre-cleared and incubated with the GFP Trap-M beads for 1 h. IPs were subjected to three washes before being lysed in 2× sample buffer and boiled. For mass spectrometry, proteins were subjected to SDS-PAGE followed by in-gel trypsin digestion. Peptide mixtures were analyzed using a Q-Exactive mass spectrometer connected to a U3000 nanoLC. Raw data was processed with MaxQuant software using an estimated 1% false discovery rate for protein identification and intensity-based absolute quantification (iBAQ) for protein quantification. For endogenous IP, mouse anti-YAP antibody (Santa Cruz Biotechnology, 63.7) was used at a concentration of 3 μg and bound to Dynabeads. Caco-2 lysates were processed as for overexpression co-IPs described above.

### EdU incorporation assay

Cells were processed for RNAi as described above (siRNA transfection) and processed with the Click-iT™ EdU Alexa Fluor™ 555 Imaging Kit (Invitrogen) according to the manufacturer's protocol.

### qPCR

Extraction of total RNA from Caco-2 siRNA-transfected cells processed as previously described (Elbediwy et al., 2016). Primers were purchased as Quantitect Primers (Qiagen).

### Antibodies

Antibodies used in mammalian cell culture were: mouse anti-GFP (clones 7.1 and 13.1, cat. no. 11814460001, Roche), mouse anti-Flag (M2, cat. no. F1804, Sigma), rabbit anti-PDLIM5 (cat. no. HPA016740, Atlas antibodies), rabbit anti-PDLIM7 (NBP1-84841, Novus), rabbit anti-YAP H-125, mouse anti-YAP (63.7) (cat. nos sc-15407 and sc-101199, Santa Cruz Biotechnology), mouse anti-α-actinin 1 (cat. no. ab18061, Abcam), rabbit anti-pY418 Src (cat. no. 44-660G, Life Technologies) and rabbit anti-Src (cat. no. 2108, Cell Signaling Technology) antibodies. Dilutions used are available from the corresponding author upon request.

### Fixation

Cells were fixed as previously described (Elbediwy et al., 2016) and were lysed in 2× sample buffer (Tris-glycine SDS containing 1× sample reducing agent; Novex).

### Mechanotransduction

Cells were plated on Prime coat substrates (2 KPa, 10 KPa and 30 KPa) (Excellness Biotech) and left for 24 h before being fixed and processed for immunofluorescence.

### siRNA transfection

siRNA transfection experiments were performed using Lipofectamine RNAiMax (Invitrogen) in Optimem and antibiotic-free medium (Gibco). Caco-2 and HEK293T cells were reverse transfected using a final concentration of 80 nM siRNA. The following day, another round of siRNA transfection was performed. Cells were left for a total of 72 h before being either fixed in PFA for immunofluorescence or lysed in sample buffer for immunoblotting. Oligonucleotides used for PDLIM5, PDLIM7, Src, Fyn, Yes and YAP were as a siGenome pool (Dharmacon).

### Inhibitor treatments

For Dasatinib experiments, cells were treated with 5 μM of the compound (Selleck chemicals) for a period of 4 h.

### Microscopy

Images were taken on a Leica SP5 laser-scanning confocal microscope.

### Quantification

For YAP localization studies, quantification was scored as one of three separate categories: N, nuclear; N/C, nuclear and cytoplasmic; and C, cytoplasmic. Cells were assessed over three independent experiments counting 500–600 cells per condition from random cellular areas. For fluorescence intensity quantification, images were measured using six independent areas of cells and over three independent experiments, and processed using ImageJ. Graphs were plotted using Prism.

## Supplementary Material

Supplementary information
