# Supplementary material for: Enigma proteins regulate YAP mechanotransduction
Source: J Cell Sci. 2018 Nov 22;131(22):jcs221788. doi: 10.1242/jcs.221788 (PMC6262774; doi:10.1242/jcs.221788)
Supplement: Supplementary information [file joces-131-221788-s1.pdf]

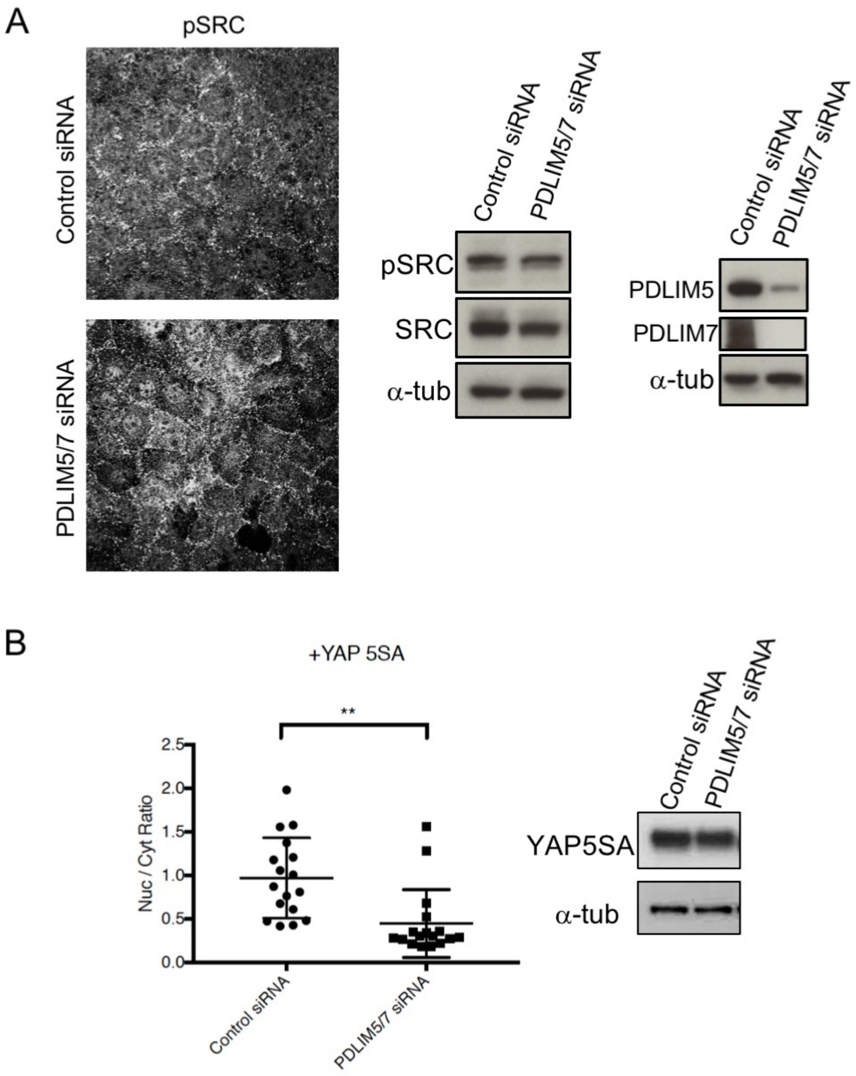

Figure S1. Enigma PDLIM5/7 family proteins are not required to regulate Src protein levels or Src auto-phosphorylation, but are required for Src to promote normal nuclear localization of YAP5SA.

(A) Silencing of both PDLIM5/7 expression in human Caco2 cells has no effect on levels of pSRC both by Immunofluorescence and Immunoblot. Note silencing efficiency is displayed on Immunoblot. (B) Silencing of PDLIM5/7 and expressing YAP5SA results in a significant decrease in the nuclear localisation of YAP as shown on graph. Protein levels of YAP5SA are unaffected on Immunoblot. ** $p < 0.01$ $n = 3$.

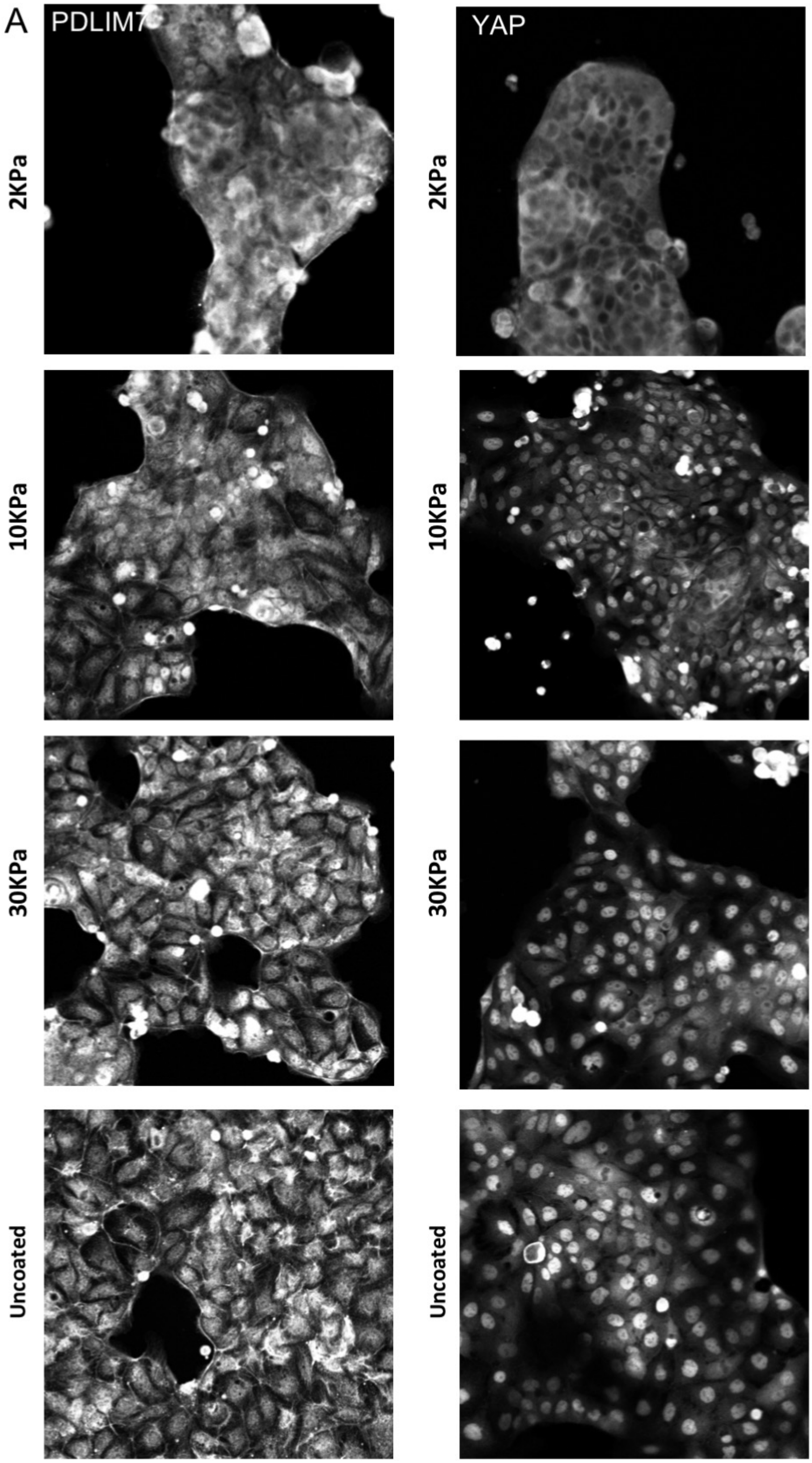

Figure S2: PDLIM7 and YAP subcellular localization are regulated by substrate stiffness.

(A) Localisation of YAP and PDLIM7 changes as hydrogel substrate stiffness increases.

Journal of Cell Science • Supplementary information

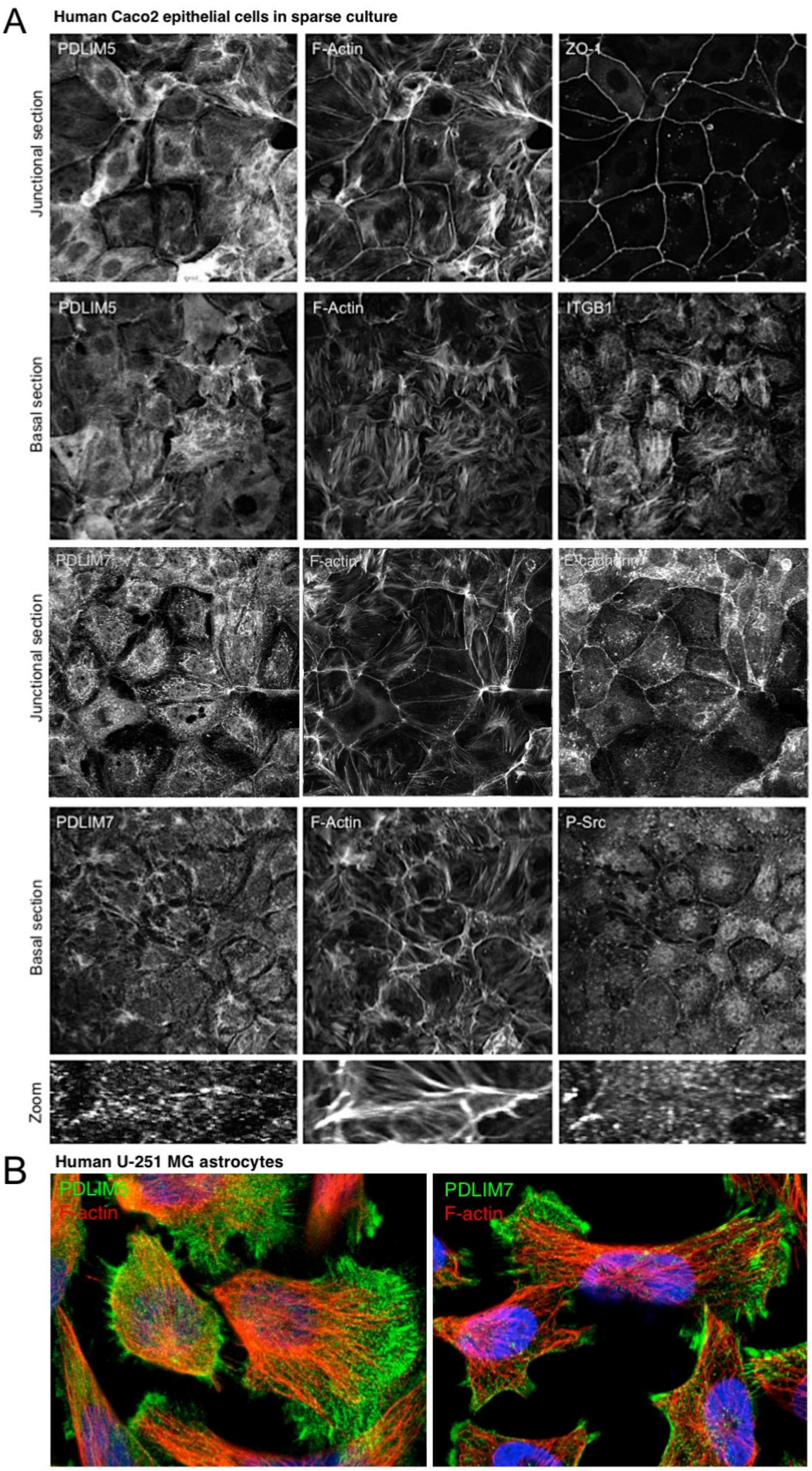

Figure S3. Localisation of Enigma PDLIM5/7 family proteins to F-actin at both Integrin adhesions and adherens junctions.

(A) Caco2 epithelial cells showing junctional and basal planes of PDLIM5 and PDLIM7 showing basal localisation of the proteins to F-actin, Integrin-beta1 and pSRC and junctional localisation to F-actin, ZO-1 and E-cadherin.

(B) U-251 astrocytes showing basal planes of PDLIM5 and PDLIM7 localizing to F-actin and focal adhesions. Adherens junctions do not form between astroctyes. Data were mined from www.proteinatlas.org.

Journal of Cell Science • Supplementary information

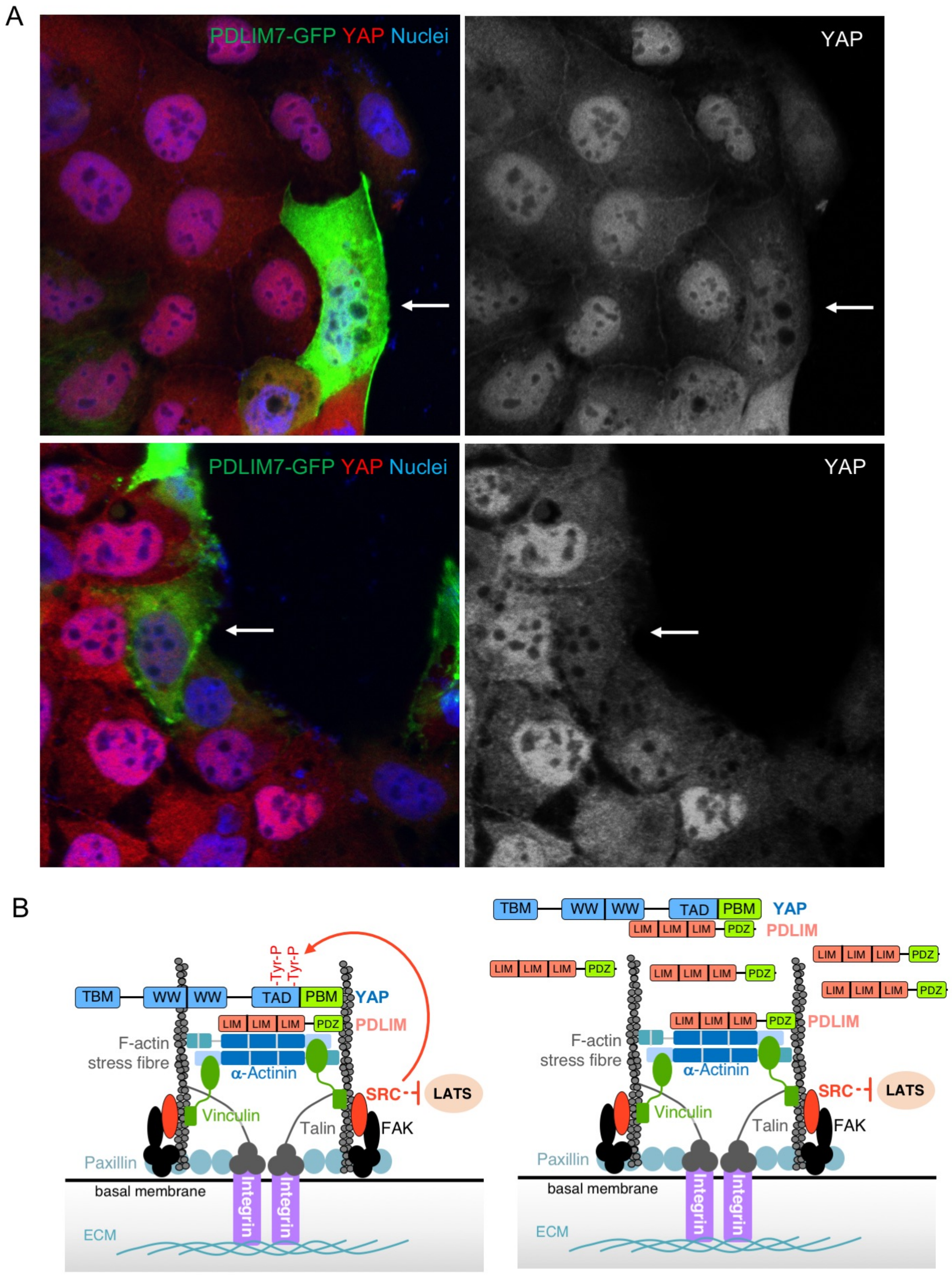

Figure S4. Overexpression of Enigman PDLIM7 causes a moderate reduction in nuclear YAP.
(A) Single flattened cells at the edge of colonies transfected with PDLIM7-GFP reveal a reduced level of YAP in the nucleus despite strong mechanical stimulation.
(B) Schematic diagram showing the possible effect of PDLIM7 overexpression in preventing YAP phosphorylation at Integrin adhesions, which is then predicted to reduce YAP nuclear localisation.

Journal of Cell Science • Supplementary information
